# Sit to stand is a new reliable method for assessing strength, power, and velocity exercise in adult pediatric cancer survivors

**DOI:** 10.1007/s00432-025-06225-7

**Published:** 2025-06-14

**Authors:** Ángela Rodríguez-Perea, Daniel Jerez-Mayorga, Esther Ubago-Guisado, Andres Marmol-Perez, Daniel Jiménez-Lupión, Andrea Rodríguez-Solana, Luis Javier Chirosa Rios, Francisco J Llorente-Cantarero, María Herrada-Robles, Luis Gracia-Marco

**Affiliations:** 1 Faculty of Physical Activity and Sports Sciences, Department of Physical and Sport Education, Universidad de León, León, Spain; 2 Department of Physical Education and Sport, Faculty of Sports Sciences, University of Granada, Granada, Spain; 3 Strength & Conditioning Laboratory, Department Physical Education and Sports, Faculty of Sport Sciences, CTS-642 Research Group, University of Granada, Granada, Spain; 4 Exercise and Rehabilitation Sciences Institute, Faculty of Rehabilitation Sciences, Universidad Andres Bello, Santiago, Chile; 5 Department of Physical Education and Sports, Faculty of Sport Sciences, Sport and Health University Research Institute (iMUDS), University of Granada, Granada, Spain; 6 Instituto de Investigación Biosanitaria ibs.Granada, Granada, Spain; 7 Instituto de Investigación Biomédica Maimonides (IMIBIC), Córdoba, Spain; 8 CIBER de Fisiopatología de la Obesidad y Nutrición (CIBEROBN), Instituto de Salud Carlos III, Madrid, Spain; 9 Departamento de Didácticas Específicas, Facultad de Ciencias de la Educación y Psicología, Universidad de Córdoba, Córdoba, Spain

**Keywords:** Resistance training, Muscle strength dynamometer, Childhood cancer, reproducibility of results, functional electromechanical dynamometer

## Abstract

**Purpose:**

We aimed to analyze the intra-set reliability of 5 sit-to-stand (5-STS) exercises with a functional electromechanical dynamometer (FEMD) and to determine and compare the load-velocity (L-V) profile in the STS exercise in adult pediatric cancer survivors by sex, age, body mass index, and type and treatment of cancer.

**Method:**

A total of 47 participants performed the 5-STS test with 5% and 20% body weight (BW) to assess intrasession reliability and analyze differences in L-V profiles by sex, age, BMI, and type and treatment of cancer.

**Results:**

Very high and extremely high relative reliability was found for both the 5% STS (ICC = 0.80–0.94) and the 20% STS (ICC = 0.87–0.95) relate to average and peak force, power, and velocity. Regarding L-V profiles, significant differences were only found in relation to sex for the velocity-axis intercept and area under the line (*p* < 0.05).

**Conclusion:**

The 5-STS test with a load of 5% and 20% of BW using a FEMD is a reliable method for assessing strength, power, and velocity exercise in adult pediatric cancer survivors. There was a relation to sex for the variables of L-V profile.

**Implications for cancer survivors:**

Reliable assessments of muscular strength, like the 5-STS test using FEMD, offer a safer, less demanding alternative to maximal strength tests (e.g., 1RM), enabling precise intensity control and better-tailored rehabilitation programs.

**Supplementary Information:**

The online version contains supplementary material available at 10.1007/s00432-025-06225-7.

## Introduction

Pediatric cancer survival has experienced a notorious increase during the last decades in developed countries with a 5-year survivorship rate over 85% (Siegel et al. 2024). However, anticancer therapies do not spare normal tissue and increase the risk of premature physiological frailty, a phenotype commonly diagnosed in geriatric population (Ness et al. 2010). The prevalence of this phenotype has been identified in up to 31.5% of adult pediatric cancer survivors and increases the risk for chronic disease and mortality (Armenian et al. 2019). Muscle function deficits is a key component of premature physiological frailty which tend to occur early after treatment completion (Marmol-Perez et al. 2024). These limitations have been identified among central nervous system tumor survivors (Ness et al. 2010). In addition, exposures such as cranial radiotherapy (Ness et al. 2007), vincristine-based chemotherapy (Ness et al. 2013) and hematopoietic stem cell transplantation (Taskinen et al. 2013), have been linked to lower-body strength and performance impairments. Specifically, childhood cancer survivors show reduced lower-extremity strength, compromised physical function (Sláma et al. 2024), limited ankle dorsiflexion, and impaired mobility (Hoffman et al. 2013; Beulertz et al. 2016). Therefore, accurate evaluation of lower-body strength is thus essential for assessing mobility and monitoring functional limitations in adult survivors of pediatric cancer.

Despite their utility, muscle strength measurements have limitations, such as variability between devices and the need for trained operators to ensure accuracy (Calixto-Lima et al. 2022). Hand dynamometers and grip strength tests have been used to evaluate muscle strength in cancer survivors (Narsale et al. 2019). In addition, lower limb impairments are often evaluated through standardized assessments such as the Timed Up & Go test and 6-minute walk test (Beulertz et al. 2016; Defeo et al. 2020). An alternative to the current methods of assessment for cancer survivors is the sit-to-stand (STS). This test has been valid and reliable to assess strength and functionality in cancer survivor patients (van Cappellen-van Maldegem et al. 2023). Various studies have used this test to identify sarcopenia in cancer survivor patients, detect frailty, estimate maximum oxygen consumption and distance walked, forecast postoperative complications, and explore the impact of chemotherapy-induced peripheral neuropathy and cancer-related fatigue on balance in different test modalities such as the 5-repetition STS (5-STS), 30-seconds STS, and 1-minute STS(Díaz-Balboa et al. 2022; Wechsler et al. 2022; van Cappellen-van Maldegem et al. 2023). However, none of these studies assessed strength directly through the STS, but rather measured the time it took to perform repetitions or the highest number of repetitions they could perform each time.

Advances in dynamometers, such as the multi-joint isokinetic dynamometer, have led to new methods for assessing functional movements and strength, power, and velocity (Dvir and Müller 2020). In addition, 5-STS has been shown to be a reliable test for assessing velocity and strength in young adults using a functional electromechanical dynamometer (FEMD) (Jerez-Mayorga et al. 2021). With the use of this technology, loads can be controlled, and thus, load-velocity (L-V) profiles can be created for a more complete assessment of patients. The L-V profile is commonly used to assess muscle function and provides information on maximum force, velocity, power, and optimal velocity (Pérez-Castilla et al. 2022). It is of interest to reproduce these profiles in research on adult pediatric cancer survivors, as measures of muscle function could allow us to detect a loss of muscle strength and power related to age, type of cancer, and type of treatment. Importantly, early identification of muscle function decline using L-V profiles could inform clinicians, allowing the implementation of targeted interventions aimed at mitigating further deterioration and potentially altering the long-term trajectory of the patients’ physical function (Jiménez-Lupión et al. 2025).

Therefore, this study aimed to analyze the reliability of 5-STS with a FEMD and to determine and compare the L-V profile in STS exercise in adult pediatric cancer survivors by sex, age, body mass index (BMI), and type and treatment of cancer.

## Methodology

### Study design

A cross-sectional study was used to assess the intra-set reliability of the 5-STS and to compare and determine the L-V profile in STS in adult pediatric cancer survivors.

### Participants

The sample recruitment was coordinated between the Andalusian School of Public Health through the Granada Cancer Registry (after cross-referencing data with the death database) and the Hematology Units of the Virgen de las Nieves Hospital in Granada. A total of 47 adult pediatric cancer survivors were included in the study (age range 18.3 to 51.6 years; 67.7 ± 14.0 kg; 166.7 ± 10.0 cm; 24.3 ± 4.3 kg/m^2^). The inclusion criteria were adults aged 18 to 55 years who had been diagnosed with pediatric cancer in the past, had been exposed to radiotherapy and/or chemotherapy, and were currently not receiving any cancer treatment. Conversely, participants were excluded if they were concurrently enrolled in other studies (whether physical, psychological, or otherwise) to prevent potential inter-study interference, or if they had a diagnosis of anorexia nervosa or bulimia, were pregnant, engaged in continuous alcohol consumption, required glucocorticoid therapy, or suffered from seasonal illnesses that could compromise study outcomes. All participants were informed of the nature of the study, aims and risks associated with the experimental procedure before giving written consent to participate. The study protocol was approved by the Institutional Review Board of the CEI- Córdoba (ref: 5131) and was conducted in accordance with the Declaration of Helsinki.

### Procedures

Participants visited the Strength and Conditioning Lab once at the University of Granada (Granada, Spain). First, anthropometric was measured, and a warm-up was performed. The warm-up included 5 min of cycling (Keiser M3i, Keiser GmbH, Germany) at a self- reported light intensity (10–40 W), 5 min of joint mobility of the lower limbs, and a specific warm-up in which the participants performed 2 sets of the unload 5-STS with a 2-min resting period between sets. Then, they performed 5-STS to familiarize themselves with the movement and explain the instructions. After the warm-up and familiarization, a field test of 5-STS was performed as fast as possible to obtain the total time. Then, familiarization with the 3-repetition STS assessment device was performed with 3 kg. The test was carried out with 5 repetitions of STS with 5% and 20% of BW with a FEMD (Myoquality M1, Myoquality Solutions, Granada. Spain)(Rodriguez-Perea et al. 2021). All assessments were carried out by two evaluators (DJ-M and DJ-L) experienced with the assessment device. Moreover, participants were asked not to perform specific strength training on the day before the assessment.

#### Anthropometric

Body mass (kg) was assessed using an electronic scale (SECA 861. Hamburg. Germany) with an accuracy of 100 g. Stature (cm) was assessed using a precision stadiometer (SECA 225, Hamburg, Germany) to the nearest 0.1 cm. BMI was calculated as body mass (kg)/height (m^2^).

#### Clinical data

Information about the type of cancer, type of treatment (radiotherapy, chemotherapy and/or surgery, alone or in combination), and time from treatment completion was obtained by self-reporting from the participants and confirmed by the hospital. In this study, cancer was classified into two categories: hematological tumors (e.g., leukemia and lymphatic system tumors) and solid tumors (e.g., sarcomas, neuroblastoma, Wilms tumor, among others).

#### Sit-to-stand

**Fig. 1:**
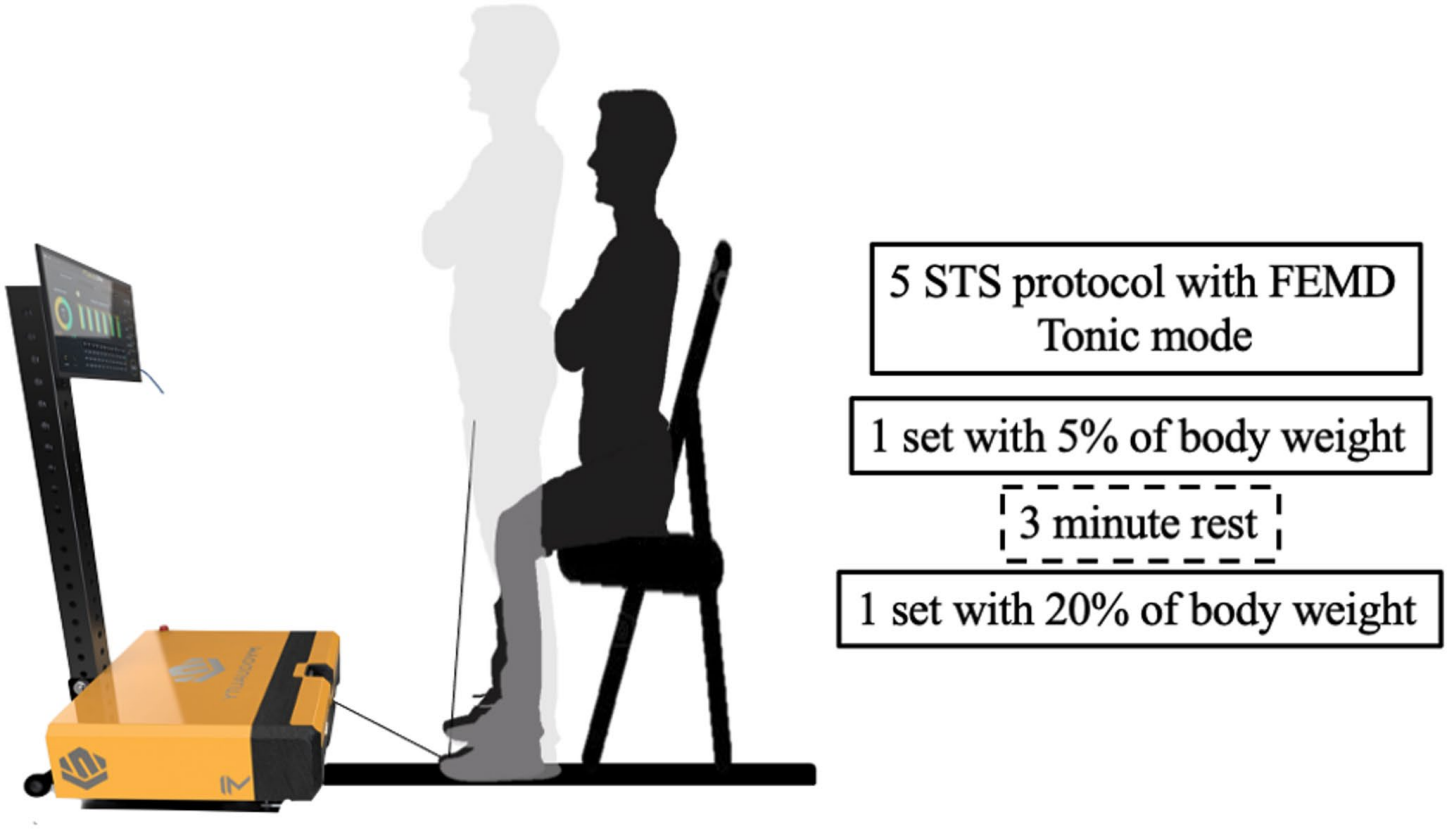
Protocol assessment of 5-STS using a functional electromechanical dynamometer (FEMD)

STS participants were seated on a rigid chair (height = 40 cm) with their arms crossed over their chest and their hip, knee, and ankle joints at approximately 90°. From this position and on the command of the countdown 3 2 1, participants were advised to perform the concentric phase as quickly as possible. A serie of 5-STS was performed with 5% BW and another serie of 5-STS with 20% BW. 3-minute rest was provided between sets. The external loads for the loaded 5-STS were determined based on the participant BW, with 5% of BW designated for the low load and 20% of BW designated for the higher load. The FEMD was programed in “Tonic Mode”, providing an external load simulating a free weight (Fig. 1).

Linear displacement was measured using the FEMD rope attached to a harness on a waistcoat used by each participant while standing up (Jerez-Mayorga et al. 2021). The mean and peak force (kg), mean and peak velocity (m·s^− 1^) and mean and peak power (W) for each repetition were recorded during the concentric phase using FEMD software. The test was considered valid if the knees were fully extended and the spine in neutral position at the end of the concentric phase and the participant sat on the chair at the end of the eccentric phase. Otherwise, the test was repeated.

### Statistical analysis

The normal distribution of all data was verified using the Shapiro-Wilk test (*p* > 0.05). The Levene’s test was used to verify the homogeneity of variances. Means ± standard deviations were calculated for all variables. Independent samples t-tests and analysis of variance were used to check whether there were significant differences between the groups. When significant differences were found, Holm–Bonferroni post hoc tests were applied to identify specific differences.

#### Reliability

Reliability was analyzed for the second and third repetitions within the 5-STS series (5% STS and 20% STS). The relative reliability was investigated using a t-test and intraclass correlation coefficient (ICC). Following Hopkins et al. (2009), we classified the magnitude of the values of the ICC through a qualitative scale: values close to 0.1 are considered low reliability; 0.3 moderate reliability; 0.5 high reliability; 0.7 very high reliability; and those close to 0.9 extremely high (Hopkins et al. 2009). We also examined the differences between the second and third repetitions using different error measures. The following methods were used to study absolute reliability: coefficient of variation (CV), standard error of measurement (SEM) as a percentage of the mean value of the measurements, and Bland-Altman plots.

Heteroscedasticity of errors was identified in the Bland-Altman plots and defined as a coefficient of determination (r^2^) > 0.1.

Finally, Cohen’s d was computed to quantify the magnitude of the difference between the test and retest. The scale used for interpreting the magnitude of the effect size (ES) was specific to training research: negligible (< 0.2), small (0.2–0.5), medium (0.5–0.8), and large (≥ 0.8)(Cohen 1988).

#### Load velocity profile

A linear model applied to the data provided by the low load (5%BW) and upper load (20%BW) was used for the mean velocity (García-Ramos et al. 2018). The trial with the mean velocity during the concentric phase (i.e., the standing phase) was used to model the L-V relationship (Garcia-Ramos and Jaric 2018). The L-V relationships were calculated by considering the mean velocity values collected under two loading conditions. A least-square linear regression model (L[V] = L_0_– sV) was used to determine the individualized L-V relationships, where L_0_ represents the load at zero velocity and s is the slope of the L-V relationship (Iglesias-Soler et al. 2021). The maximal velocity capacity (V_0_) and the area under the L-V relationship line (A_line_) were calculated as follows: V_0_ = L_0_/s and A_line_ = L_0_ ⋅ V_0_/2.

We conducted analyses for the whole sample, as well as separately by sex (male/female), age (young adults < 35 years; adults ≥ 35 years), BMI (under/normal weight < 25.00 kg/m^2^; overweight/obesity ≥ 25.00 kg/m^2^), type of cancer (hematological or solid), and treatment of cancer (chemotherapy and radiotherapy, non-chemotherapy, and non-radiotherapy) for all analyses.

All analyses were performed using the Statistical Package for Social Sciences (IBM SPSS Statistics for Windows. version 28.1; Armonk. NY) and the level of significance was set at *p* < 0.05.

## Results

The descriptive characteristics of the participants and differences according to sex, age, BMI, and type and treatment of cancer are presented in Table 1. Table 2 summarizes the distribution of cancer types among the study cohort. Cancers were classified as hematological tumors (leukemias, lymphomas, histiocytosis) or solid tumors (thyroid carcinoma, sarcomas, Wilms tumor, brain tumors, and other miscellaneous solid tumors). Percentages represent the proportion of each type relative to the total sample. The mean and peak force, power, and velocity of the two loads (5%BW and 20%BW STS) and the absolute and relative reliability in the second and third repetitions are shown in Table 3. These percentages of BW corresponded to loads ranging from 2 kg to 6 kg (for the 5%BW) and from 7 kg to 26 kg (for the 20%BW). No significant differences were found in any variable (*p* > 0.05) and the effect size of the mean difference was less than 0.08. Very high and extremely high relative reliability was found for both the 5%STS (ICC = 0.80–0.93) and 20%STS (ICC = 0.86–0.96) in average and peak force, power, and velocity. The 20% STS condition (ICC > 0.86; CV < 19.74) obtained higher reliability values than the 5% STS condition (ICC > 0.80; CV < 36.99). The Bland-Altman plots are shown in Supplementary Figs. 1 and 2. Heteroscedasticity of error was observed in the mean and peak force and peak power of 5%STS (*r* > 0.1).

**Table 1:** Descriptive characteristics of the sample and differences according to sex, age, BMI, type of cancer, and type of treatment groups

Participants (*n*)	Age (years)	Weight (kg)	Stature (cm)	BMI (kg/m^2^)	Time of diagnostic (years)	5%STS Load (kg)	20%STS Load (kg)
Whole sample (47)	33.7 ± 9.0	67.7 ± 14.0	166.7 ± 10.0	24.3 ± 4.3	8.9 ± 4.4	3.5 ± 0.7	13.7 ± 2.8
Male (25)	32.0 ± 9.4	72.6 ± 14.9	172.3 ± 8.7	24.4 ± 4.2	8.2 ± 4.1	3.7 ± 0.8***	14.6 ± 3.0
Female (22)	35.5 ± 8.3	62.1 ± 10.8	160.3 ± 7.3	24.3 ± 4.6	9.8 ± 4.9	3.1 ± 0.5	12.6 ± 2.2
Young adults (23)	26.2 ± 5.7***	68.2 ± 14.2	170.9 ± 9.8**	23.2 ± 3.8*	8.0 ± 4.6	3.5 ± 0.7	13.9 ± 2.7
Adults (24)	40.8 ± 4.7	67.3 ± 14.1	162.7 ± 8.7	25.4 ± 4.7	9.9 ± 4.3	3.4 ± 0.7	13.5 ± 2.9
Under/Normal weight (31)	32.2 ± 8.4*	60.2 ± 7.8***	166.7 ± 10.3	21.6 ± 1.7***	8.5 ± 4.7	3.1 ± 0.4***	12.2 ± 1.5***
Overweight/Obesity (16)	36.4 ± 9.6	82.2 ± 11.8	166.6 ± 9.7	29.5 ± 2.9	10.0 ± 4.0	4.1 ± 0.7	16.6 ± 2.5
Hematological (21)	31.6 ± 8.4	67.5 ± 13.8	166.6 ± 10.9	24.3 ± 4.2	8.8 ± 3.6	3.5 ± 0.7	13.8 ± 2.6
Solid (26)	35.3 ± 9.2	67.9 ± 14.4	166.8 ± 9.5	24.3 ± 4.5	9.1 ± 5.2	3.4 ± 0.8	13.6 ± 3.0
Chemotherapy and radiotherapy (18)	36.1 ± 7.3*^b^	67.0 ± 15.4	165.4 ± 10.2	24.3 ± 4.5	7.1 ± 4.9*^a^	3.5 ± 0.7	13.7 ± 3.0
Non-chemotherapy (9)	38.8 ± 8.6^*c^	62.6 ± 10.7	164.1 ± 9.1	23.1 ± 2.7	12.0 ± 2.9*^c^	3.1 ± 0.6	12.6 ± 2.4
Non-radiotherapy (20)	29.2 ± 8.7	70.7 ± 13.9	169.0 ± 10.2	24.9 ± 4.9	9.3 ± 4.0	3.6 ± 0.8	14.2 ± 2.8

Data are presented as mean ± standard deviation. BMI = body mass index; STS = sit-to-stand. Significance level *p < 0.05 **p < 0.01 *** *p* < 0.001; ^a^ Differences between chemotherapy and radiotherapy with non-chemotherapy, ^b^Differences between chemotherapy and radiotherapy with non-radiotherapy, ^c^Differences between non-chemotherapy with non-radiotherapy

**Fig. 2:**
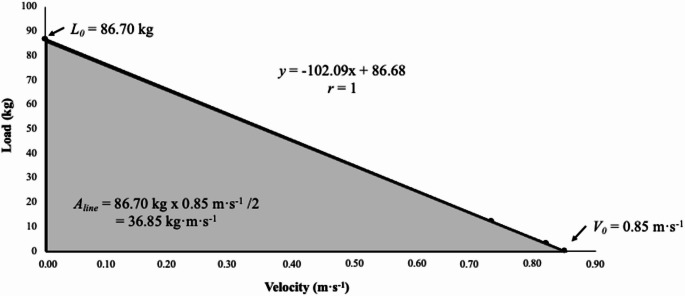
Representation of the load-velocity relationship obtained from a representative participant during the STS exercise using 5% and 20% BW. The regression equation and Pearson’s product-moment correlation coefficient *(r)* are shown. *L*_*0*_, load-axis intercept; *V*_*0*_, velocity-axis intercept; *A*_*line*_, area under the line

Figure 2 shows the representation of the L-V relationship obtained by a representative participant during the STS exercise using 5% BW and 20% BW. The difference in variables derived from the L-V profile obtained during the STS and divided by groups are shown in Table 4. Significant differences (*p* < 0.05) were only found in relation to sex for the variables *V*_*0*_ and *A*_*line*_.

**Table 2:** Distribution of cancer types by category of survivors included in this study

Type of Cancer	Category	*N*	% (of total)
Leukemia	Hematological tumor	11	23.4%
Lymphoma (all types)	Hematological tumor	9	19.1%
Histiocytosis	Hematological tumor	1	2.1%
Thyroid carcinoma (papillary, follicular, papillotubular)	Solid tumor	5	10.6%
Sarcomas (Ewing/Ewing-like sarcoma, osteosarcomas)	Solid tumor	6	12.8%
Wilms tumor	Solid tumor	3	6.4%
Brain tumors (including polar spongioblastoma)	Solid tumor	3	6.4%
Germ cell tumors (testicular, ovarian dysgerminoma, immature teratoma)	Solid tumor	3	6.4%
Neuroblastoma	Solid tumor	1	2.1%
Retinosblastoma	Solid tumor	1	2.1%
Pancreatic tumor	Solid tumor	1	2.1%
Intestinal neoplasm	Solid tumor	1	2.1%

**Table 3:** Intraset reliability of STS (mean ± SD)

		Test (*n* = 47)	Retest (*n* = 47)	*p*-value	Cohen’s d	ICC (95% CI)	% CV	SEM
Mean Force (kg)	5% BW	4.1 ± 1.6	4.2 ± 1.9	0.749	0.03	0.81 (0.69–0.89)	18.87	0.78
	20% BW	16.7 ± 6.1	16.6 ± 5.8	0.767	-0.02	0.86 (0.77–0.92)	13.62	2.27
Peak Force (kg)	5% BW	12.3 ± 6.7	12.9 ± 9.2	0.402	0.08	0.80 (0.67–0.88)	29.00	3.65
	20% BW	41.0 ± 20.5	41.4 ± 20.9	0.641	0.02	0.95 (0.92–0.97)	11.32	4.66
Mean Power (W)	5% BW	32.9 ± 25.3	32.9 ± 27.0	0.972	0.00	0.88 (0.80–0.93)	27.83	9.16
	20% BW	125.1 ± 92.5	122.6 ± 91.8	0.603	-0.03	0.93 (0.88–0.96)	19.74	24.45
Peak Power (W)	5% BW	104.3 ± 91.5	108.9 ± 110.4	0.566	0.05	0.85 (0.76–0.92)	36.99	36.41
	20% BW	331.9 ± 254.4	333.9 ± 247.5	0.856	0.01	0.96 (0.92–0.98)	15.98	53.21
Mean Velocity (m·s^− 1^)	5% BW	0.746 ± 0.28	0.730 ± 0.26	0.300	-0.06	0.93 (0.88–0.96)	9.92	0.07
	20% BW	0.692 ± 0.30	0.680 ± 0.31	0.427	-0.04	0.94 (0.89–0.96)	11.38	0.08
Peak Velocity (m·s^− 1^)	5% BW	1.469 ± 0.43	1.449 ± 0.44	0.454	-0.05	0.91 (0.85–0.95)	8.93	0.13
	20% BW	1.388 ± 0.47	1.391 ± 0.48	0.906	0.01	0.94 (0.90–0.97)	8.47	0.12

ICC = intraclass correlation coefficients; CI = confident interval; SEM = standard error of measurement; %CV = percentage coefficient of variation; BW = body weight

**Table 4:** Difference in variables derived from the load velocity profile obtained during STS and divided by groups

		L_0_ (kg)	Slope	V_0_ (m·s^− 1^)	A_line_ (kg·m·s^− 1^)
Total	Total (*n* = 47)	122.65	138.75	0.897	59.33
Sex	Male (*n* = 25)	140.99	140.36	0.957*	93.87*
	Female (*n* = 22)	101.81	136.91	0.829	40.28
Age	Young adults (*n* = 23)	139.00	165.55	0.846	65.14
	Adults (*n* = 24)	106.98	113.06	0.946	53.76
BMI	Under/Normal weight (*n* = 31)	125.06	143.25	0.873	59.45
	Overweight and obesity (*n* = 16)	117.99	130.03	0.944	59.09
Type of cancer	Hematological tumor (*n* = 21)	142.63	150.38	0.923	72.23
	Solid tumors (*n* = 26)	106.52	129.35	0.876	48.90
Type of treatment	Chemotherapy and radiotherapy (*n* = 18)	160.94	182.90	0.888	78.57
	Non-chemotherapy (*n* = 9)	89.80	105.65	0.916	40.55
	Non-radiotherapy (*n* = 20)	102.98	113.90	0.897	50.46

L_0_ = load-axis intercept; V_0_ = velocity-axis intercept; *A*_*line*_ = area under the line. Significance level *p < 0.05

## Discussion

This study aimed to analyze the reliability of 5-STS with a FEMD and to determine and compare the L-V profile in STS exercise in adult pediatric cancer survivors by sex, age, BMI, and type and treatment of cancer. The integration of STS exercise and L-V profile analysis may be beneficial for the assessment and monitoring of the physical abilities of adult survivors of pediatric cancer, contributing to their rehabilitation and overall well-being. The main findings of our study show that the 5-STS test with 5% BW and 20% BW assessed with FEMD proved to be a reliable test for evaluating mean and peak force, power, and velocity in adult survivors of pediatric cancer. Moreover, the L-V profiles in this population were determined, revealing a notable difference in mechanical power (*A*_*line*_*)* and velocity (*V*_*0*_) between males and females, with higher values observed in males. This discrepancy is associated with a reduced risk of falls, enhanced functional capacity, and lower mortality risk (Jiménez-Lupión et al. 2023).

To the best of our knowledge, this is the first study to analyze the reliability of STS in adult pediatric cancer survivors and to create L-V profiles with STS in this population using motorized technology for electronic load control.

### Reliability of STS

Cancer survivors experience a reduction in muscle mass and consequently a decrease in strength, functionality, and quality of life (Neyroud et al. 2021). Therefore, it is crucial to develop valid and reliable tests to assess muscular strength. A previous study determined the reliability of the strength and movement velocity of the concentric phase from 5-STS using three incremental loads measured by FEMD in healthy young adults and obtained a high reliability for the three loads in terms of strength and velocity (ICC > 0.87; CV < 10.67%). This study, like our study, conducted evaluations in tonic mode but did not establish individualized loads; instead, they set three different loads (Jerez-Mayorga et al. 2021). Additionally, in this study, intersession reliability was analyzed, whereas in our study, intrasession reliability was assessed along with power evaluation (Jerez-Mayorga et al. 2021). We obtained high absolute reliability for both mean power and peak power, with better reliability observed when the participants were evaluated with 20% BW.

Previous studies have demonstrated the validity of an encoder in measuring the power of the STS test to measure the functional and morphological level of older women (Lindemann et al. 2015) and to assess fatigue through the loss of velocity (Lindemann et al. 2016). Another study, conducted by Gray and Paulson (2014) demonstrated the reliability of the Tendo LPT Weightlifting Analyzer device for measuring power in the 10-repetition STS test. Another study by Ruiz-Cárdenas et al. (2018) demonstrated the validity and reliability of the STS of an iPhone App for measuring time, velocity, and power in the performance of a single repetition of STS.

### L-V profiles

Once we tested the reliability of 5-STS with FEMD, L-V profiles were obtained for cancer survivors. Previous studies have performed L-V profiles in breast cancer survivors using the bench press (Franco-López et al. 2024) and leg extension exercises (Díez-Fernández et al. 2023). A study by Franco-López et al. (2024) showed that female breast cancer survivors could use the same velocity parameters derived from the L-V profile as healthy women. Díez-Fernández et al. (2023) recommended using the two-point method to evaluate bilateral leg press 1RM in breast cancer survivors to monitor training intensity. Another study used the STS test to validate velocity load and velocity force profiles in older adults with an isokinetic device and demonstrated moderate reliability for strength and power but not for velocity (Piche et al. 2021). However, no studies have created L-V profiles across different types of cancer. In this study, we analyzed L-V profile variables according to cancer type, including patients with hematological tumor (such as leukemia, lymphoma, and histiocytosis) and solid tumors (such as thyroid carcinoma, sarcomas, Wilms tumor, brain tumors, germ cell tumors, neuroblastoma, retinoblastoma, pancreatic tumors, and intestinal neoplasms).

Compared with maximal strength assessment methods such as the one-repetition maximum (1RM) test, the L-V profile provides an alternative approach for evaluating muscle function. Although 1RM and L-V are distinct methods, both aim to assess strength capabilities. The L-V profile is physically less demanding, less influenced by motivational factors, and reduces the risk of injury (Bochicchio et al. 2023), making it especially suitable for vulnerable populations such as cancer survivors. In these individuals, functional strength training often targets lower extremity muscles necessary for movements such as walking, rising from a chair, and climbing stairs (Rock et al. 2023). Furthermore, evaluating the L-V profile allows for individualized and objective load control, improving training quality and outcomes (González-Badillo and Sánchez-Medina 2010). In many studies on cancer survivors, such as that by Rock et al. (2023), training intensity has been adjusted based on subjective perception rather than objective load measures. Understanding the L-V profile can therefore enhance exercise prescription by tailoring loads to each patient’s capacity, potentially leading to more significant functional improvements.

In our study, the only difference found in the variables related to the L-V profile was a function of sex, with lower mechanical power (A_line_) and velocity (V_0_) in females. Previous investigations have shown that female survivors of childhood cancer have a higher risk of deterioration of health status, which is consistent with the results of our study (Hudson et al. 2003; Oeffinger et al. 2006). A recent study evaluated the force-velocity profile in older adults and compared variables related to the force-velocity profile between males and females. Similar to our study, significant differences in power and velocity were found in the four loads evaluated, being significantly higher in male participants (Bochicchio et al. 2023). Because muscle strength is a variable that influences health, these quick and low-cost assessments could be performed to evaluate strength in female cancer survivors. Accurate measurements of muscle strength are critical for personalization of exercise programs in cancer survivors that maximize functional recovery and improve quality of life (Calixto-Lima et al. 2022).

On the other hand, various investigations have found a relationship between muscle mass or strength and aspects of medical care, such as the frequency of hospitalizations, duration of treatment and rehabilitation outcomes, highlighting the potential economic implications of muscle strength assessments in the care of pediatric cancer survivors (Bruyère et al. 2019; Cruz-Jentoft et al. 2019). For instance, in a study conducted by Rodwin et al. (2021), neuromuscular dysfunction was found to be prevalent among childhood cancer survivors, with its incidence increasing post-treatment, and being associated with poorer health and socioeconomic outcomes. Neyroud et al. (2021) emphasized the diagnostic and therapeutic challenges related to cancer, associated muscle loss and dysfunction, underlining the importance of early detection. Therefore, implementing accessible and cost-effective tools to assess and improve muscle strength, deficits in strength and power production, such as FEMD or functional strength test, could play a critical role in the long-term and quality of life of adult pediatric cancer survivors.

### Strengths and limitations

Typically, characterizing the L-V relationship requires two to four weight conditions, resulting in differences in at least 0.5 m·s^− 1^ between the lightest and heaviest loads. However, considering adult pediatric cancer survivors, we opted not to employ excessively high loads or significantly fatigue the participants, thus using loads of 5% BW and 20% BW. Nevertheless, these loads did not fully represent the velocity loss criteria, and higher loads are recommended to determine L-V profiles. However, this study has provided a new reliable test to assess muscle strength in adult survivors of pediatric cancer and has created and compared L-V profiles based on age, sex, BMI, cancer type, and type of treatment received.

## Conclusion

In conclusion, findings from the present study demonstrated that assessing muscle strength using the 5-STS test with FEMD in adult survivors of pediatric cancer is a reliable and valuable practice, with implications for clinical care, rehabilitation, and healthcare resource utilization. The determination of L-V profiles allows for the improvement of training focused on enhancing muscle strength in adult survivors of pediatric cancer.

## Electronic supplementary material

Below is the link to the electronic supplementary material.

Supplementary Material 1

Supplementary Material 2

## Data Availability

No datasets were generated or analysed during the current study.
