# Supplementary material for: Sit to stand is a new reliable method for assessing strength, power, and velocity exercise in adult pediatric cancer survivors
Source: J Cancer Res Clin Oncol. 2025 Jun 14;151(6):189. doi: 10.1007/s00432-025-06225-7 (PMC12166015; doi:10.1007/s00432-025-06225-7)
Supplement: Supplementary file 1 — Supplementary Material 1 [file 432_2025_6225_MOESM1_ESM.docx]

**
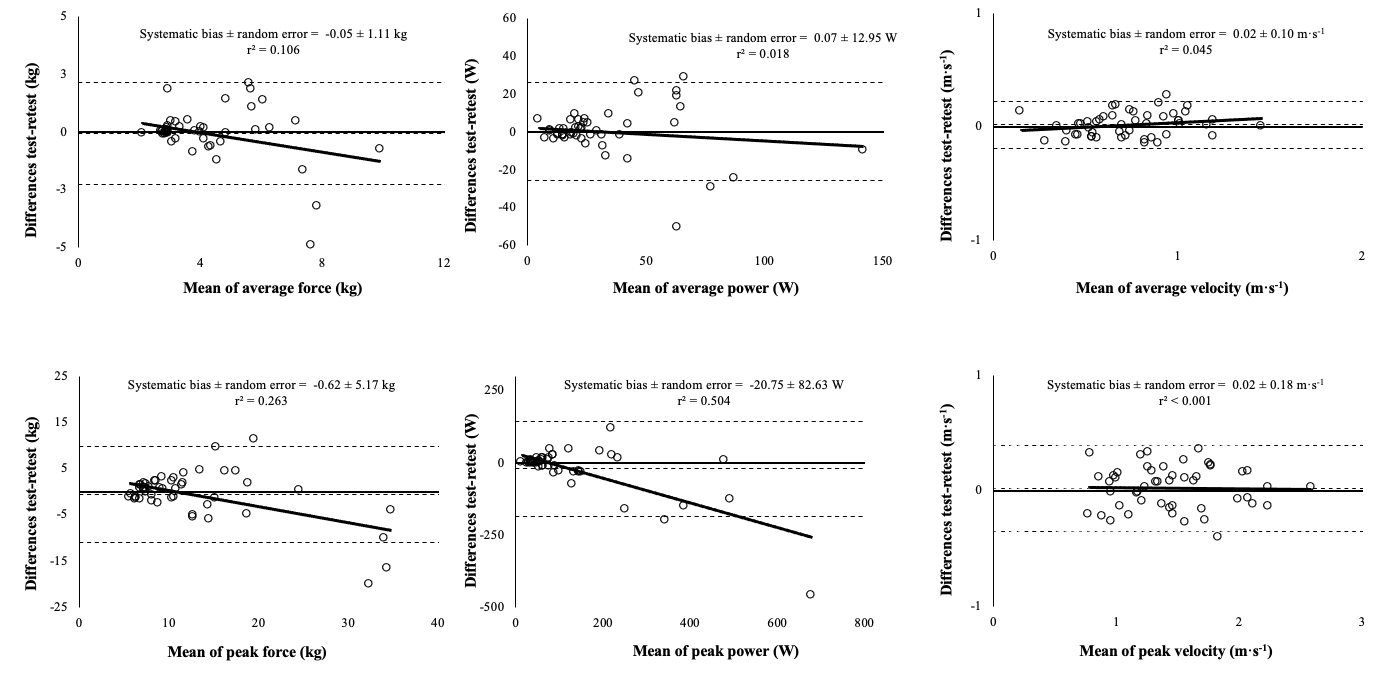
**

**Supplementary Figure 1.** The Bland-Altman plots depict the average difference between the second and third repetitions of 5% STS and 95% limits of agreement (dashed lines). along with the regression line (solid line). In addition. a solid line indicates the 0 point.

**
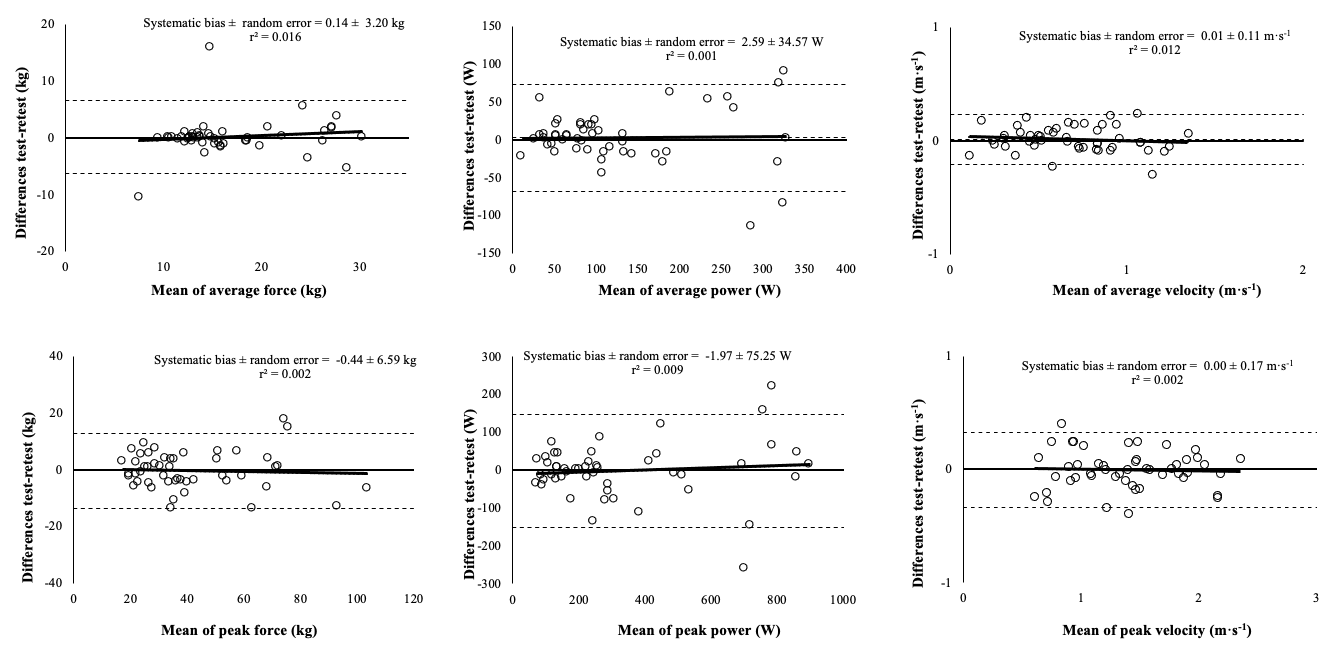
**

**Supplementary Figure 2.** The Bland-Altman plots depict the average difference between the second and third repetitions of 20% STS and 95% limits of agreement (dashed lines) along with the regression line (solid line). In addition. a solid line indicates the 0 point.
