# Supplementary figures and images for: Sit to stand is a new reliable method for assessing strength, power, and velocity exercise in adult pediatric cancer survivors
Source: J Cancer Res Clin Oncol. 2025 Jun 14;151(6):189. doi: 10.1007/s00432-025-06225-7 (PMC12166015; doi:10.1007/s00432-025-06225-7)

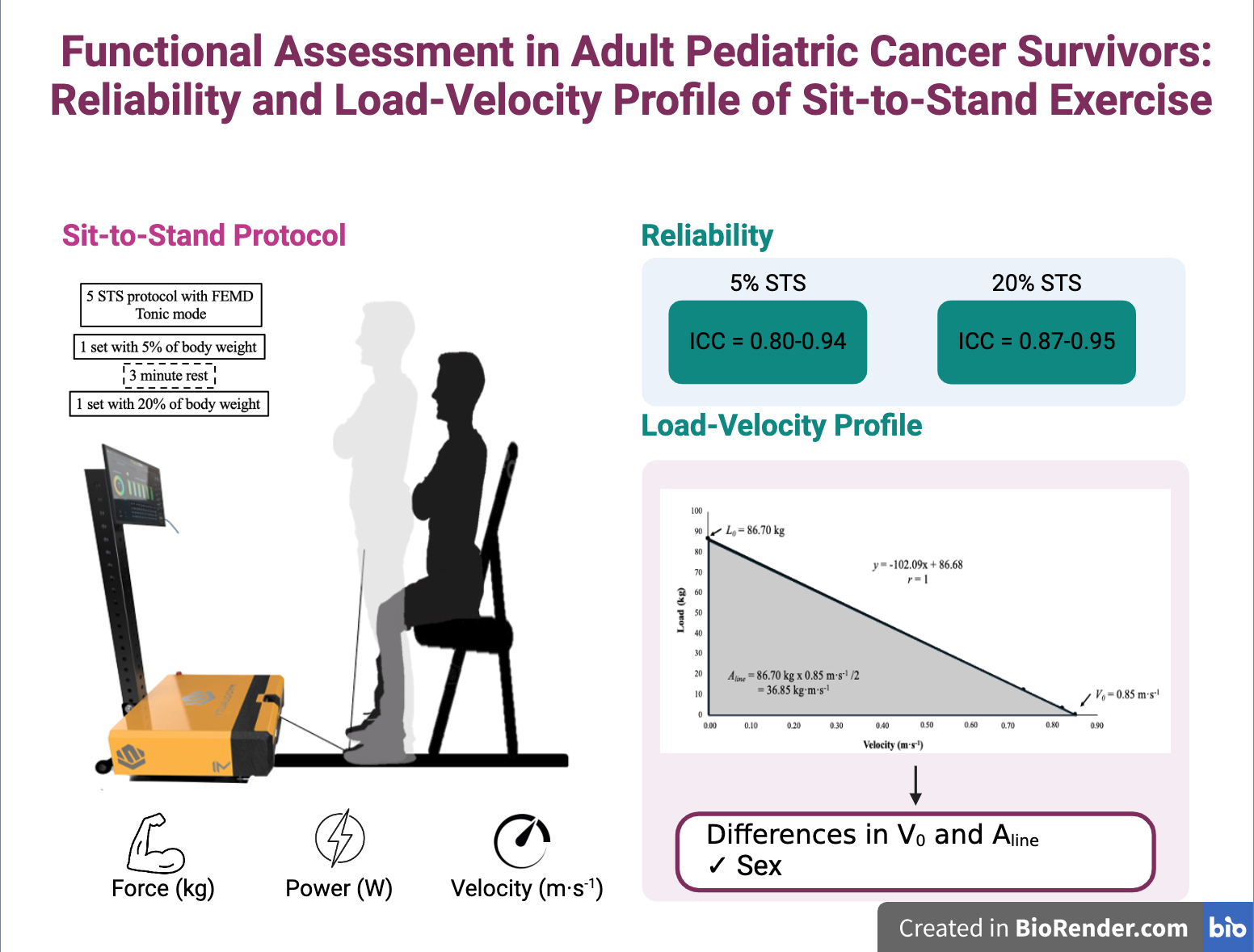

Supplement: Supplementary file 2 — Supplementary Material 2 [file 432_2025_6225_MOESM2_ESM.png]
